# Pneumatosis Intestinalis in a Patient with Acute Promyelocytic Leukemia

**DOI:** 10.1155/2015/576042

**Published:** 2015-10-08

**Authors:** Abhishek Mangaonkar, Joshua Mansour, Ryan Keen, Tarun Kukkadapu, Rohini Chintalapally, Vamsi Kota

**Affiliations:** ^1^Department of Internal Medicine, Medical College of Georgia, Augusta, GA 30912, USA; ^2^Department of Hematology/Oncology, Winship Cancer Institute of Emory University, Atlanta, GA 30322, USA

## Abstract

Pneumatosis Intestinalis is a rare condition characterized by the presence of gas within the intestinal wall. We describe a case of a 33-year-old woman with acute promyelocytic leukemia who developed nausea and nonbloody diarrhea. CT showed intramural air in transverse and descending colon. Patient clinically improved with conservative management.

## 1. Introduction

Acute promyelocytic leukemia (APL) is a subtype of acute myeloid leukemia characterized by a balanced reciprocal translocation between chromosomes 15 and 17 [1]. It is a highly curable leukemia with cure rates greater than 90% in most cooperative group trials. However, population-based studies indicate significant early complications, with up to 30% early deaths [2], most common causes being bleeding and differentiation syndrome.

Pneumatosis Intestinalis (PI) is a rare condition characterized by the presence of gas within mucosal and submucosal layers of the intestinal wall [3]. It is a radiographic finding and an abdominal film may show a change in characteristic of the intestinal wall in two-thirds of patients. However, one-third of patients require a CT Scan to confirm diagnosis. CT is more sensitive and may help detect additional findings that may point out the etiology [4]. PI is an unusual finding that can be benign in some conditions, but in severe cases it could be life-threatening and require surgical intervention.

Two major theories exist regarding the etiology of this disease and the formation of these colonic cystic collections of air. According to the mechanical theory, an increase in bowel luminal pressure allows for gas to penetrate the submucosal space into lymphatic channels. This can occur secondary to bowel obstruction, trauma, surgery, and colonoscopy [5]. The bacterial theory suggests that gas-producing bacteria such as* Clostridium* or* Escherichia Coli* invade the mucosal layers and produce gas, which is retained in these regions [6].

Treatment should be based on clinical presentation as well as underlying etiology. Medical and conservative management is generally used unless patient does not respond or emergent surgery is required secondary to peritonitis, perforation, and abdominal sepsis [7].

## 2. Case

Patient is a 33-year-old woman who presented to an outside hospital after a two-week history of cough and shortness of breath where she was found to be severely anemic with hemoglobin of 4.0, a platelet count of 20,000, and a peripheral smear showing blasts and schistocytes. The patient was then transferred to our institution for further evaluation. A bone marrow biopsy was performed, which confirmed APL. The patient was then started on ATRA 50 mg twice daily and idarubicin 24 grams every 48 hours. As she was at a high risk for differentiation syndrome with an elevated WBC count of >10 on initiation of therapy, dexamethasone 10 mg twice daily was started.

On day 12 of admission, the patient indicated that she had persistent, but improving nausea. She later had developed nonbloody watery diarrhea for 2 days.* Clostridium difficile* PCR and stool studies for parasites and ova were negative. On day 14 of therapy the patient complained of new onset left lower quadrant pain in addition to her diarrhea. Vital signs at that time were stable with patient being afebrile, borderline hypertensive, and mildly tachycardiac and respiratory rate was within normal limits. On physical examination, abdomen was soft, nondistended, and diffusely tender. Lactic acid was elevated at 3.9 mmol/L.

**Figure 1:**
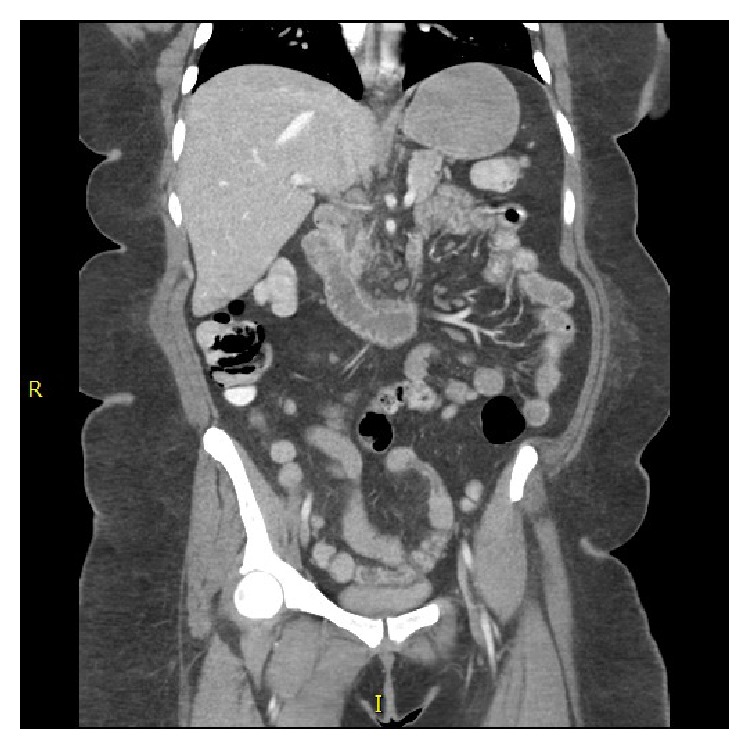
Showing Pneumatosis Intestinalis in right lower quadrant in a coronal cut of CT imaging.

**Figure 2:**
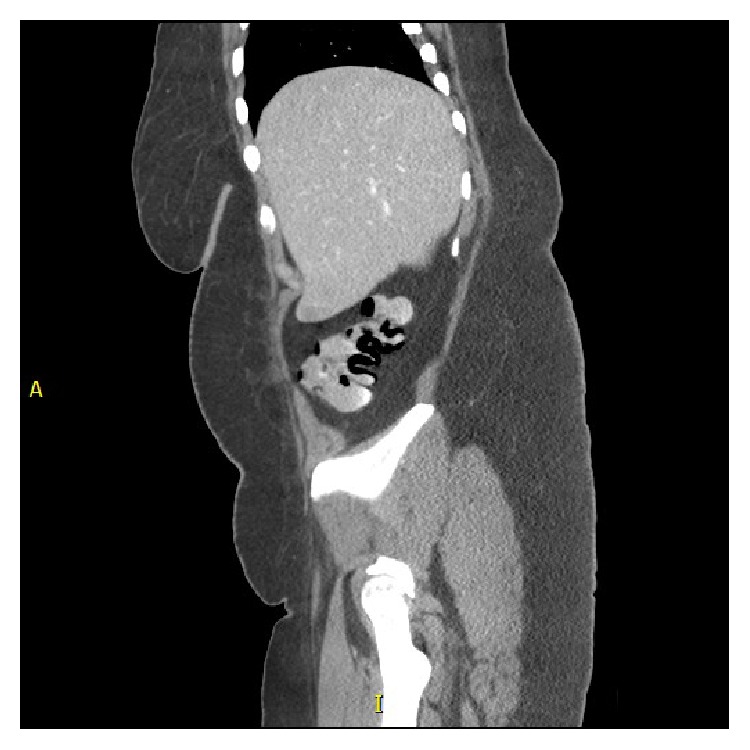
Showing Pneumatosis Intestinalis in right lower quadrant in a sagittal cut of CT imaging.

A CT of the abdomen and pelvis with oral and IV contrast was performed given patient's symptoms. The small bowel demonstrated subtle enhanced inflammatory changes of the jejunum, normal ileum, and fluid distention to the level of the terminal ileum, where there was Pneumatosis Intestinalis into the cecum. There was again focal Pneumatosis Intestinalis in the mid transverse colon with colonic mesenteric venous gas and focal pneumatosis of the distal transverse colon, as well as the distal descending colon. There was no free intraperitoneal air to suggest viscous perforation (Figures 1 and 2). However, given the results of the CT Scan an emergent consult to GI surgery was placed.

**Figure 3:**
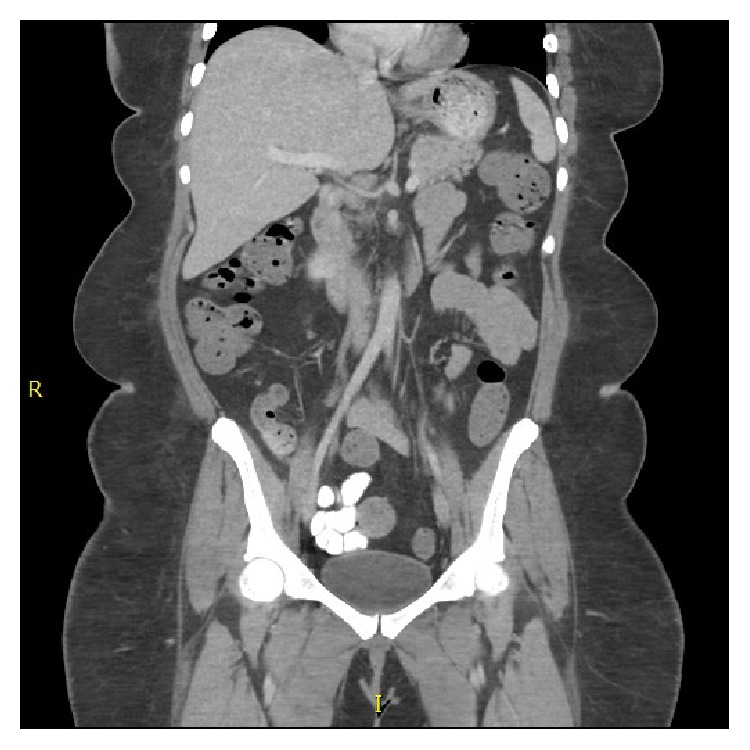
Showing resolution of Pneumatosis Intestinalis on CT imaging.

GI surgery recommended no emergent surgical intervention at the time and continuation with serial abdominal exams overnight. Both the oncology and surgery teams continually monitored the patient by trending lactic acid, observing new onset bloody diarrhea and abdominal pain. Patient was started on metronidazole and ciprofloxacin for bowel inflammation and prophylaxis. Eventually, patient did not require an urgent surgical intervention and PI resolved within 3 weeks with conservative management (Figure 3). After the initial induction with ATRA and idarubicin, she was treated with two cycles of arsenic consolidation as per the Cancer and Leukemia Group B (CALGB) regimen. Her initial diagnosis was in April 2013. Further, patient relapsed in June 2014 while being on maintenance therapy with ATRA, 6-mercaptopurine, and methotrexate. She was re-treated with ATRA, arsenic, dexamethasone, and hydroxyurea. Subsequently, she went into remission till May 2015 when she had another relapse detected by cerebrospinal fluid analysis. She is currently being treated with ATRA/ATO and intrathecal methotrexate. Since the initial episode, she did not have any further episodes of PI.

## 3. Discussion

There is no clinical finding characteristic of PI as patients have a diverse clinical presentation from being asymptomatic to having symptoms such as abdominal distension, diarrhea, nausea, vomiting, and rectal bleeding [8]. Patients with cancer are especially at high risk due to immunosuppressive therapies, most notably corticosteroids and chemotherapy [9].

Furthermore, it has been proposed that long-term administration of corticosteroids in patients undergoing chemotherapy, postallogeneic stem cell transplant patients, or patients with chronic COPD may possibly induce erosion of the intestinal mucosa, leading to lymphoid depletion and mucosal disturbances causing penetration of gas into the submucosal layer [9–11]. In these patients, the depletion of Peyer's patches in the distal ileum and colon has also been hypothesized as a cause of benign PI [10, 12–14].

Benign PI is often an incidental benign finding in cancer patients, found on follow-up CT imaging. However, it is important to differentiate between benign and life-threatening disease as some patients may require urgent surgical intervention. Patients should be categorized based on whether they can be medically managed, who may benefit from surgery, who must undergo surgery, and in whom surgery may do more harm than good. Categorization is important as mortality rate in patients who need surgery depends on the time-interval between diagnosis and intervention [15]. Greenstein et al. further showed that patients above 60 years of age with one or more symptoms of obstruction (emesis, nausea, and pain), a WBC >12,000/mm^3^, and portomesenteric venous gas on CT imaging should be considered for surgery.

In conclusion, it is important to have a high index of clinical suspicion to diagnose PI in APL. Etiology of PI is often multifactorial and it is difficult to identify a definite cause in patients with multiple comorbidities, such as malignancy and chronic immunosuppression.

## References

[1] Larson R. A., Kondo K., Vardiman J. W., Butler A. E., Golomb H. M., Rowley J. D. (1984). Evidence for a 15; 17 translocation in every patient with acute promyelocytic leukemia. *The American Journal of Medicine*.

[2] Powell B. L., Moser B., Stock W. (2010). Arsenic trioxide improves event-free and overall survival for adults with acute promyelocytic leukemia: north American Leukemia Intergroup Study C9710. *Blood*.

[3] Ejtehadi F., Chatzizacharias N. A., Kennedy H. (2012). Pneumatosis intestinalis as the initial presentation of systemic sclerosis: a case report and review of the literature. *Case Reports in Medicine*.

[4] Azzaroli F., Turco L., Ceroni L. (2011). Pneumatosis cystoides intestinalis. *World Journal of Gastroenterology*.

[5] Galandiuk S., Fazio V. W. (1986). Pneumatosis cystoides intestinalis: a review of the literature. *Diseases of the Colon and Rectum*.

[6] Suzuki H., Murata K., Sakamoto A. (2009). An autopsy case of fulminant sepsis due to pneumatosis cystoides intestinalis. *Legal Medicine*.

[7] St. Peter S. D., Abbas M. A., Kelly K. A. (2003). The spectrum of pneumatosis intestinalis. *Archives of Surgery*.

[8] Knechtle S. J., Davidoff A. M., Rice R. P. (1990). Pneumatosis intestinalis. Surgical management and clinical outcome. *Annals of Surgery*.

[9] Lee K. S., Hwang S., Rúa S. M. H., Janjigian Y. Y., Gollub M. J. (2013). Distinguishing benign and life-threatening pneumatosis intestinalis in patients with cancer by CT imaging features. *American Journal of Roentgenology*.

[10] Laskowska K., Burzyńska-Makuch M., Krenska A. (2012). Pneumatosis cystoides interstitialis: a complication of graft-versus-host disease. A report of two cases. *Polish Journal of Radiology*.

[11] Freeman H. J. (2014). Spontaneous free perforation of the small intestine in adults. *World Journal of Gastroenterology*.

[12] Pieterse A. S., Leong A. S.-Y., Rowland R. (1985). The mucosal changes and pathogenesis of pneumatosis cystoides intestinalis. *Human Pathology*.

[13] Shin D.-K., Oh J., Yoon H., Kim J. E., Chong S. Y., Oh D. (2012). Asymptomatic pneumatosis intestinalis following chemotherapy for B lymphoblastic leukemia with recurrent genetic abnormalities in an adolescent patient. *Korean Journal of Hematology*.

[14] Khalil P. N., Huber-Wagner S., Ladurner R. (2009). Natural history, clinical pattern, and surgical considerations of pneumatosis intestinalis. *European Journal of Medical Research*.

[15] Greenstein A. J., Nguyen S. Q., Berlin A. (2007). Pneumatosis intestinalis in adults: management, surgical indications, and risk factors for mortality. *Journal of Gastrointestinal Surgery*.

